# Closing Remarks for the Special Issue on Coronaviruses and Influenza Viruses: Evolution, Cross-Species Transmission, and Recombination

**DOI:** 10.3390/v18080894

**Published:** 2026-08-13

**Authors:** Maged Gomaa Hemida

**Affiliations:** Department of Veterinary Biomedical Sciences, Lewyt College of Veterancy Medicine, Long Island University, 720 Northern Blvd., Brookville, NY 11548, USA; maged.hemida@liu.edu

The completion of this Special Issue, “Coronaviruses and Influenza Viruses: Evolution, Cross-Species Transmission, and Recombination”, offers a valuable opportunity to reflect on the remarkable scientific progress in understanding two of the most critical groups of emerging and re-emerging viral pathogens that affect both human and animal health [1]. The collection of articles published here highlights the dynamic evolutionary nature of coronaviruses and influenza viruses. It underscores the critical role of genomic surveillance, molecular epidemiology, virus–host interactions, and One Health approaches in mitigating future pandemic threats. The COVID-19 pandemic fundamentally changed our understanding of the risks posed by zoonotic viruses. It demonstrated how rapidly a novel pathogen can emerge, adapt to a new host, spread globally, and have profound impacts on public health, economies, and societies [2]. Similarly, influenza viruses continue to evolve through antigenic drift, antigenic shift, reassortment, and adaptation to new host species, remaining persistent threats to global health. The coexistence of these viral pathogens across humans, domestic animals, wildlife, and the environment creates numerous opportunities for viral evolution, recombination, and cross-species transmission, highlighting the need for continuous vigilance and scientific innovation [3]. The contributions assembled in this Special Issue collectively underscore the central role of genomic surveillance in monitoring viral evolution and emergence [1,4]. Several studies demonstrated how molecular epidemiological investigations can reveal the introduction, circulation, diversification, and replacement of viral variants over time. These findings reinforce the value of next-generation sequencing technologies and large-scale genomic monitoring programs in providing early warning signals for emerging variants and informing public health interventions. The increasing accessibility of sequencing technologies and bioinformatics tools has transformed our ability to track viral evolution in near real time, allowing researchers and policymakers to make evidence-based decisions during outbreaks and epidemics [5].

Another major theme emerging from this Special Issue is the importance of cross-species transmission and host adaptation. Coronaviruses and influenza viruses possess exceptional capacities to infect multiple host species and occasionally overcome species barriers. Understanding the molecular determinants that facilitate host switching remains one of the most important challenges in modern virology. The studies presented in this issue contribute valuable insights into the complex interactions between viruses and their hosts and further support the concept that many future emerging infectious diseases are likely to originate at the human–animal environment interface. These observations strengthen the scientific foundation for implementing comprehensive One Health surveillance strategies that integrate human, veterinary, and environmental health sectors [6]. Recombination, reassortment, and genetic diversification also emerged as recurring themes throughout the Special Issue. Coronaviruses possess a remarkable ability to undergo homologous recombination, while influenza viruses frequently generate novel variants through reassortment events. These evolutionary mechanisms contribute significantly to viral adaptation, immune escape, host range expansion, and the emergence of strains with altered pathogenicity. Continued monitoring of these processes is essential for anticipating future outbreaks and improving preparedness strategies [7,8]. The studies included in this collection provide important evidence that viral evolution remains an ongoing and dynamic process requiring sustained global surveillance efforts. Beyond surveillance and evolutionary analyses, the Special Issue highlights the importance of advancing diagnostic technologies, antiviral strategies, and vaccine development. The rapid evolution of RNA viruses presents ongoing challenges for maintaining the effectiveness of existing diagnostics and vaccines. Future efforts should focus on developing broadly protective and universal vaccine platforms capable of providing durable protection against diverse viral variants. In parallel, innovative antiviral approaches targeting conserved viral and host factors may offer additional tools for mitigating the impact of future outbreaks. Looking forward, several emerging technologies have the potential to transform the field of virology. Artificial intelligence and machine learning approaches are increasingly being applied to predict viral evolution, identify critical mutations, discover novel therapeutic targets, and accelerate vaccine design. Similarly, advances in systems biology, structural biology, synthetic biology, and computational modeling provide unprecedented opportunities to better understand virus–host interactions and improve outbreak preparedness [9,10,11,12,13]. The integration of these technologies with traditional virological approaches will likely play a central role in future efforts to combat emerging viral diseases. As Guest Editor, I would like to express my sincere gratitude to all authors who contributed their high-quality research, to the reviewers whose expertise ensured the scientific rigor of the published manuscripts, and to the editorial staff of Viruses for their outstanding support throughout the publication process [14]. The success of this Special Issue reflects the collective efforts of an international community of researchers dedicated to advancing our understanding of viral evolution, transmission, and pathogenesis.

In conclusion, this Special Issue illustrates both the substantial progress made and the significant challenges that remain in the study of coronaviruses and influenza viruses. Continued interdisciplinary collaboration, international data sharing, genomic surveillance, and One Health research initiatives will be essential to addressing future emerging infectious disease threats. We hope the findings presented in this collection will stimulate further scientific discoveries, foster new collaborations, and contribute to the development of innovative strategies to protect human and animal health worldwide.
